# Editorial: Public health challenges in post-Soviet countries during and beyond COVID-19

**DOI:** 10.3389/fpubh.2023.1290910

**Published:** 2023-10-10

**Authors:** Natalya Glushkova, Yuliya Semenova, Antonio Sarria-Santamera

**Affiliations:** ^1^Department of Epidemiology, Biostatistics and Evidence Based Medicine, Al-Farabi Kazakh National University, Almaty, Kazakhstan; ^2^School of Medicine, Nazarbayev University School of Medicine, Astana, Kazakhstan

**Keywords:** COVID-19, healthcare, health services accessibility, delivery of health care, public health administration, post-Soviet countries

The dissolution of the Union of Soviet Socialist Republics (USSR) on December 25, 1991, marked a monumental event in the latter half of the 20th century. It led to the emergence of fifteen former socialist republics—Armenia, Azerbaijan, Belarus, Estonia, Georgia, Kazakhstan, Kyrgyzstan, Latvia, Lithuania, Moldova, Russia, Tajikistan, Turkmenistan, Ukraine, and Uzbekistan—as independent nations, breaking away from the former union. This significant dissolution triggered a profound socio-economic crisis, the repercussions of which endured for many years. In fact, some of these independent states have yet to fully recover their pre-dissolution levels of socio-economic development, even as of today (1). Currently, the wealthiest post-Soviet states are those that possess ample natural resources, such as Russia, Kazakhstan, Uzbekistan, Turkmenistan, and Azerbaijan, and those like the three Baltic states that successfully transitioned to market-oriented economies and established stable political systems. The objective of this paper is to describe the public health situation in those countries.

During the Soviet era, the Semashko model proved to be both cost-effective and well-structured, significantly contributing to the overall health improvement of the Soviet population. This centralized, all-encompassing healthcare model featured a hierarchical structure, with the state funding and providing healthcare services to its citizens free of charge. It placed a particular emphasis on controlling communicable diseases such as typhus and tuberculosis, with primary and hospital care as its cornerstones. The Semashko model led to several improvements, ensuring the availability of essential vaccines and achieving high vaccination coverage rates, as well as low rates of infant and maternal mortality (2).

However, in the 1980s, the collapse of oil prices, upon which the USSR's economy heavily relied, initiated a decline in healthcare quality due to insufficient investment and mismanagement. By the time of the USSR's dissolution, the healthcare system was grappling with shortages of basic equipment, medications, and modern technologies, despite having an abundant supply of healthcare professionals. Consequently, the former Soviet republics inherited an underfunded and inefficient healthcare system, compelling them to confront the numerous challenges arising from the union's dissolution (3). Following the disintegration of the USSR, the newly independent nations were compelled to undertake comprehensive reforms of their public systems, including healthcare, which had long adhered to the Semashko system.

After the dissolution of the USSR, many countries initiated healthcare system reforms that led to the privatization of state-owned health facilities. The empirical evidence of the impact of those reforms is very limited, but seems that has not contributed to an improvement in outcomes (4). However, due to chronic underfunding and a lack of political commitment, post-Soviet countries generally retain many aspects of the Semashko model, resulting in shared similarities in the overall structure and governance of healthcare systems, characterized by a strong vertical hierarchy (3).

National Ministries of Health (MoH) play a central role in healthcare governance. They are responsible for designing and implementing health policies and introducing legislative changes. These MoHs not only handle planning but also oversee healthcare provision, with minimal delegation of decision-making to subnational health authorities. Public involvement in health policy formulation is lacking, and patient rights remain inadequately protected. Moreover, there is still over-reliance on large hospitals, and the allocation of health resources lacks transparency (5). Only a few countries have introduced mandatory health insurance, with more planning to implement it in the future. However, private and voluntary health insurance is largely non-existent, primarily due to the low-income levels in the general population, making it challenging for many to afford such coverage. Furthermore, out-of-pocket payments constitute a significant share of healthcare expenditures, exceeding 60% in Armenia, Tajikistan, and Turkmenistan (6), which is well-above the level recommended by the World Health Organization (WHO) of 20% (7). This predisposes people to catastrophic health expenditures in a situation of a public health emergency.

Given that the majority of healthcare professionals were (and still are) low-paid salaried employees, and due to the shortage of funds in the healthcare sector, and insufficient regulatory oversight, medical corruption became a matter of concern. Meanwhile, corruption has a significant negative impact on healthcare systems and the health outcomes of the population. As such, it was estimated that countries with high levels of corruption in the healthcare sector have twice as high childhood and infant mortality rates as compared to the countries with low rates of medical corruption (8).

The trend of underfunding in healthcare persists to this day. According to World Bank data from 2020, only Armenia allocated healthcare expenditures at a level typical of many developed nations, at 12.24% of the Gross Domestic Product (GDP). In contrast, other former Soviet countries allocated significantly less, with Kazakhstan, one of the region's industrial leaders, spending only 3.79% of GDP on healthcare (6).

Many national governments implemented a series of health plans aimed at strengthening primary healthcare, improving the accessibility of medical services, and modernizing hospital infrastructure. These plans placed significant emphasis on the control of both infectious and non-communicable diseases (NCDs). Similar to many other nations, NCDs are the leading cause of mortality, significantly contributing to lower life expectancy compared to European Union (EU) levels. Premature mortality is a significant factor, with the highest rates observed in men, often exceeding a 10-year gender gap. Cardiovascular disease (CVD) ranks as the primary cause of death, followed by oncological and digestive system disorders. Several factors contribute to this high rate of premature mortality, including excessive alcohol consumption, smoking, and an unhealthy diet. Poor control of arterial hypertension and dyslipidemia further contributes to premature mortality from conditions like ischemic heart disease, stroke, and hypertensive heart disease (Azfar et al.). In some countries, governments have made significant investments in tertiary prevention of CVD, making it accessible and affordable. However, secondary prevention is suboptimal, and primary prevention strategies are lacking. To bridge these gaps, a multi-pronged approach is necessary. There is a need for the prioritization of primary CVD prevention with a focus on the reduction of modifiable risk factors and the promotion of healthier foods, as well as encouraging more regular physical exercise, given the shift toward a more sedentary lifestyle in many post-Soviet nations. Concurrently, efforts to enhance the management of arterial hypertension and dyslipidemia for secondary prevention should be intensified.

When it comes to cancer, the second most common cause of mortality among NCDs, lung cancer takes the top spot in the majority of post-Soviet nations. This prevalence is attributed to the high rates of smoking and ambient air pollution resulting from industrial and traffic emissions (Zhylkybekova et al.). In contrast, stomach cancer is the primary cause of cancer-related mortality in post-Soviet countries with lower levels of economic development, such as Tajikistan, Kyrgyzstan, and Uzbekistan. This can be attributed to factors such as a high prevalence of Helicobacter pylori infection, overcrowded living conditions, and the influence of certain environmental factors (Albuquerque et al.). Furthermore, there is a growing trend of breast cancer, which has become the most common cancer site among females (Midlenko et al.). Addressing this trend requires the implementation of public health strategies for control and prevention. There is a need to make cancer screening programs accessible and affordable for the people, as well as to implement the “best-buy” campaigns to increase the uptake of the existing cancer screening programs.

Historically, the USSR had robust infection control services, including disease surveillance. However, after its dissolution, infection control significantly deteriorated, especially during the early transition period. As a result, new challenges like HIV/AIDS and multidrug-resistant tuberculosis (MDR-TB), have emerged. During the 1990s and early 2000s, there was a significant increase in TB incidence and mortality, which started declining only in the second half of the 2000s. By the end of the 2010s, most post-Soviet nations achieved a substantial decline in TB mortality, except for Russia, where it remains elevated due to high rates of heavy alcohol intake, incarceration, and the burden of HIV/AIDS (9). In general, during the 1990s and early 2000s, post-Soviet nations bore witness to one of the world's most rapidly expanding HIV/AIDS epidemics, along with a surge in cases of viral hepatitis B and C, primarily due to the widespread use of injection drugs. While the prevalence of injection drug use has decreased over the past decade, several post-Soviet countries, including Russia and Turkmenistan, continue to prohibit buprenorphine or methadone substitution therapy, despite its effectiveness. This presents a persistent challenge in managing the HIV epidemic in these regions. Moreover, with evolving patterns of HIV transmission, the coverage of anti-HIV programs remains inadequate, and there is a notable deficiency in the availability of antiviral treatments, which heavily relies on external funding sources (Gabdullina et al.).

Another pressing public health concern in the region is antibiotic resistance, highlighting the imperative need for action (10).

The COVID-19 pandemic exposed the existing gaps in infection control. Despite the fact that many post-Soviet countries share borders with China, the first cases of COVID-19 were not registered until February-March of 2020. Following these initial cases, countries immediately implemented strict measures, including lockdowns, curfews, and border closures between regions. As the COVID-19 epidemic progressed, countries faced a severe shortage of hospital infrastructure and medical professionals (Gazezova et al.). The rapid initial increase in the number of COVID-19 cases overwhelmed the healthcare system, leading to waiting lists for emergency hospitalization. This was partly due to a previous reduction in the number of hospital beds (11). Countries with significant financial resources were able to mobilize them in an effort to contain the epidemic. However, even these resources were insufficient to meet the rapidly growing demands for medicines, equipment, diagnostic tests, and hospital facilities.

With the introduction of the Sputnik-V vaccine in 2020, Russia has emerged as a significant COVID vaccine producer. Consequently, the geopolitical affiliations of this major country within the post-Soviet space exert an influence on public health dynamics and contribute to enhanced healthcare management in affiliated countries. Despite the early availability of anti-COVID-19 vaccines, post-Soviet countries have exhibited some of the lowest vaccination rates due to a high prevalence of vaccine hesitancy (Peshkovskaya et al.). This reluctance to get vaccinated can be attributed to the tense relations between governments and citizens, a common feature of the former USSR. As a result, the general public lacks trust in state institutions, including the healthcare system and science. It was surprising to observe that many healthcare professionals shared these views, occasionally discouraging patients from getting vaccinated and assisting them in avoiding vaccination (Adambekov et al.). To address the issue of vaccine hesitancy, special strategies need to be devised, focusing on immunization policy, capacity building, addressing population fears, and promoting positive behavior change.

Although the health of the population in post-Soviet countries began to improve at the beginning of the 21st century, the COVID-19 crisis resulted in excess mortality, a decrease in average life expectancy, and exposed many unresolved issues in healthcare system management and financing (12). To address these existing gaps, further healthcare reforms need to be planned. For these reforms to succeed, the participation of all major stakeholders is necessary, including government officials, the medical community, and patient representatives (13). Additionally, decentralization of government structures is required to enhance governance effectiveness. This process must be accompanied by the allocation of sufficient resources and an increase in healthcare system financing. The allocation of healthcare resources should be fair and transparent, with efforts made to involve the population in healthcare management. There is a need to increase the professionalism and improve qualification of healthcare managers and administrators (14).

Given that the former Soviet Union represents one of the world's largest geographical regions with low population density, its public health challenges differ from those in other regions. Therefore, the introduction of biomedical technologies related to digitalization, biosensors, and similar advancements might offer greater cost-effectiveness. Importantly, the process of digitizing the healthcare system must continue, as many post-Soviet countries currently lack comprehensive health information systems (15). Additionally, there is a need to shift focus from administratively monitoring outputs to identifying relevant outcomes, analyzing potential origins or impacts of poor performance, and implementing ambitious quality improvement programs (16). Aside from the introduction of advanced technologies, political stability could substantially contribute to a better healthcare model.

Academic institutions can play a pivotal role by providing education to strengthen the public health workforce and conducting research to generate new evidence. In addition, academia can serve as a cornerstone for reform and improvement, actively engaging with governments in the formulation of policies, providing expert insights, and addressing healthcare challenges. Academic institutions can promote public awareness and participation by organizing public health campaigns, disseminating health information, and involving the community in healthcare decision-making. Collaboration between academia, government, and healthcare organizations is essential to achieve sustainable and positive change in the healthcare landscape of post-Soviet nations.

## Author contributions

YS: Writing—review and editing. NG: Writing—original draft. AS-S: Writing—review and editing.
